# Adsorption and self-assembly of porphyrins on ultrathin CoO films on Ir(100)

**DOI:** 10.3762/bjnano.11.134

**Published:** 2020-10-05

**Authors:** Feifei Xiang, Tobias Schmitt, Marco Raschmann, M Alexander Schneider

**Affiliations:** 1 Solid State Physics, Friedrich-Alexander-Universität Erlangen-Nürnberg (FAU), 91058 Erlangen, Germany

**Keywords:** adsorption energy, molecular rotors, porphyrins, self-assembly, transition metal oxides

## Abstract

Porphyrins represent a versatile class of molecules, the adsorption behavior of which on solid surfaces is of fundamental interest due to a variety of potential applications. We investigate here the molecule–molecule and molecule–substrate interaction of Co-5,15-diphenylporphyrin (Co-DPP) and 2*H*-tetrakis(*p*-cyanophenyl)porphyrin (2H-TCNP) on one bilayer (1BL) and two bilayer (2BL) thick cobalt oxide films on Ir(100) by scanning tunneling microscopy (STM) and density functional theory (DFT). The two substrates differ greatly with respect to their structural and potential-energy landscape corrugation with immediate consequences for adsorption and self-assembly of the molecules studied. On both films, an effective electronic decoupling from the metal substrate is achieved. However, on the 1BL film, Co-DPP molecules are sufficiently mobile at 300 K and coalesce to self-assembled molecular islands when cooled to 80 K despite their rather weak intermolecular interaction. In contrast, on the 2BL film, due to the rather flat potential landscape, molecular rotation is thermally activated, which effectively prevents self-assembly. The situation is different for 2H-TCNPP, which, due to the additional functional anchoring groups, does not self-assemble on the 1BL film but forms self-assembled compact islands on the 2BL film. The findings demonstrate the guiding effect of the cobalt oxide films of different thickness and the effect of functional surface anchoring.

## Introduction

Due to their variability with respect to functionalization and corresponding potential applications, porphyrins represent a fascinating class of molecules the properties of which at inorganic interfaces still have to be fully understood [1]. The investigation of adsorption properties and structures using surface science methods has been very successful in recent decades, but it mainly focused on metal or semi-metal surfaces. The research revealed numerous ways to steer self-assembly and on-surface reactions by a careful choice of functionalization and substrates [2]. Despite its importance for applications, the investigation of the structure and binding of porphyrins on semiconducting and, notably, metal oxide surfaces remains scarce [3–13]. We chose here to investigate two different porphyrins on thin films of cobalt(II) oxide (CoO). Cobalt(II) oxide not only is a semi-conducting oxide, it is also an anti-ferromagnet due to electron correlation effects and it shows catalytic activity [14–17]. As a thin film grown on Ir(100), the oxide is of extremely high quality [18–20] avoiding the complexity that arises from atomic-scale defects in bulk materials [5–8]. The atomic structure of these films has been investigated in detail by diffraction methods [18–21]. The corresponding data ensures reproducibility of the cobalt oxide preparation and meaningful computer-based substrate modeling.

Functionalization of porphyrins may introduce new properties of the molecular adlayer, for example, by introducing elements that allow for strong anchoring [8,10–13]. This may, however, counteract the possibility to form ordered layers when, due to the enhanced molecule–substrate interaction, the molecules become immobile or the functional groups used as anchors cannot give rise to intermolecular forces anymore. The molecules employed in this study, Co-5,15-diphenylporphyrin (Co-DPP) and 2*H*-tetrakis(*p*-cyanophenyl)porphyrin (2H-TCNPP), are representatives with which this important hierarchy of interaction energies may be studied. While Co-DPP anchors mainly through its central metal atom and provides rather weak intermolecular interaction via its phenyl substituents, 2H-TCNPP may use its cyanophenyl groups for either coupling to the substrate or for providing relatively strong molecule–molecule interactions. The two molecules have been investigated on metal surfaces. It was shown that free-base (2H) or metalated DPP molecules assemble in two-dimensional or linear structures on metal surfaces [22–23] and that on-surface dehydrogenative polymerization reactions may be induced [24]. DPP molecules interact via the phenyl rings and the molecular macrocycle. In contrast, 2H-TCNPP is found to either form metal-organic networks or structures that, depending on the substrate, are stabilized by hydrogen bonding or dipolar coupling via its cyano groups [10,25–26]. This is similar to findings for other cyano-functionalized molecules [27–30]. The aim of this paper is to find the adsorption geometry of the two molecules, to demonstrate electronic decoupling from the supporting Ir substrate, and to reveal the relevant energetic hierarchy that may lead to self-assembly on the particular oxide surfaces.

## Experimental

**Figure 1:**
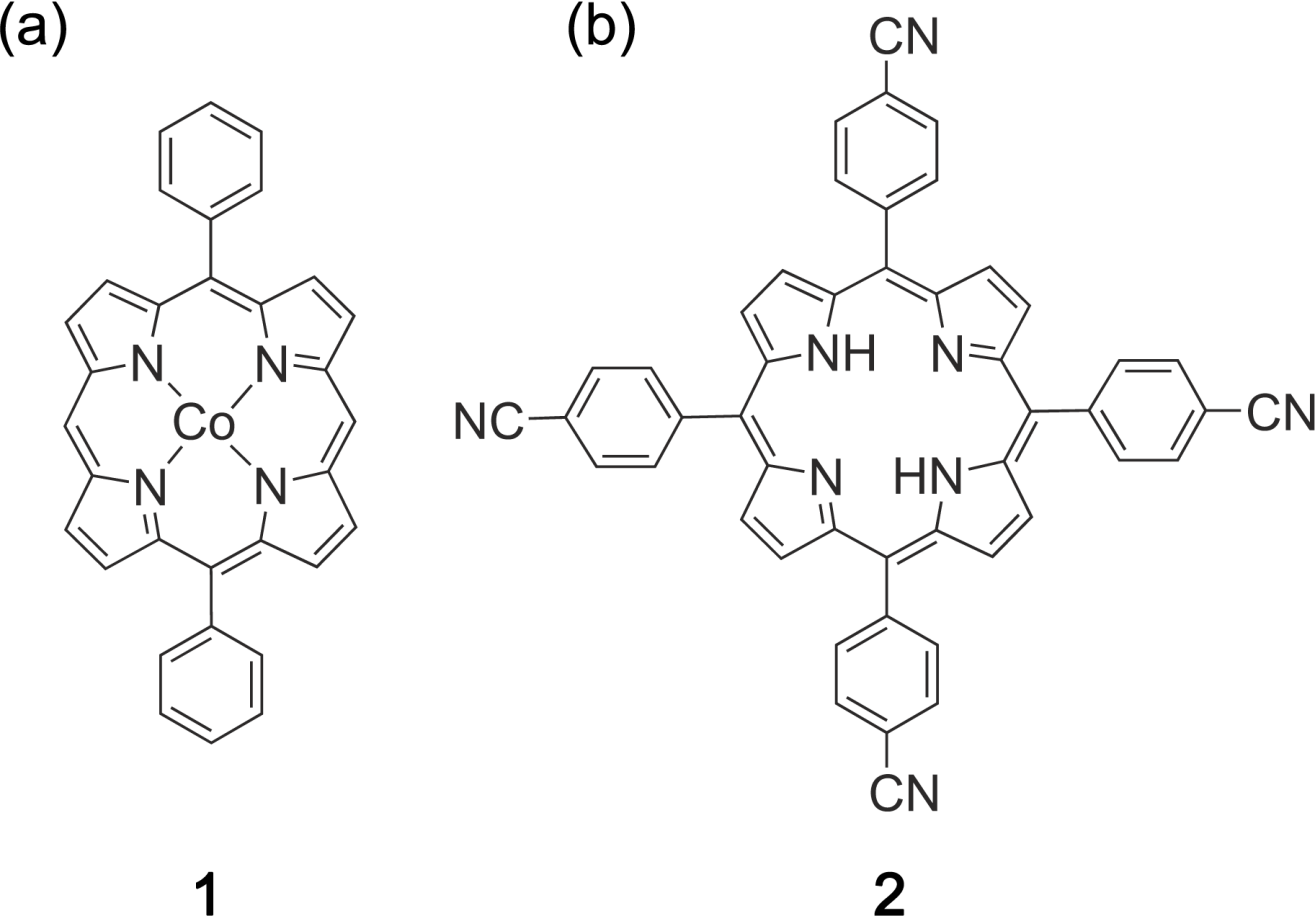
Chemical structure of (a) Co-DPP (**1**) and (b) 2H-TCNPP (**2**).

**Figure 2:**
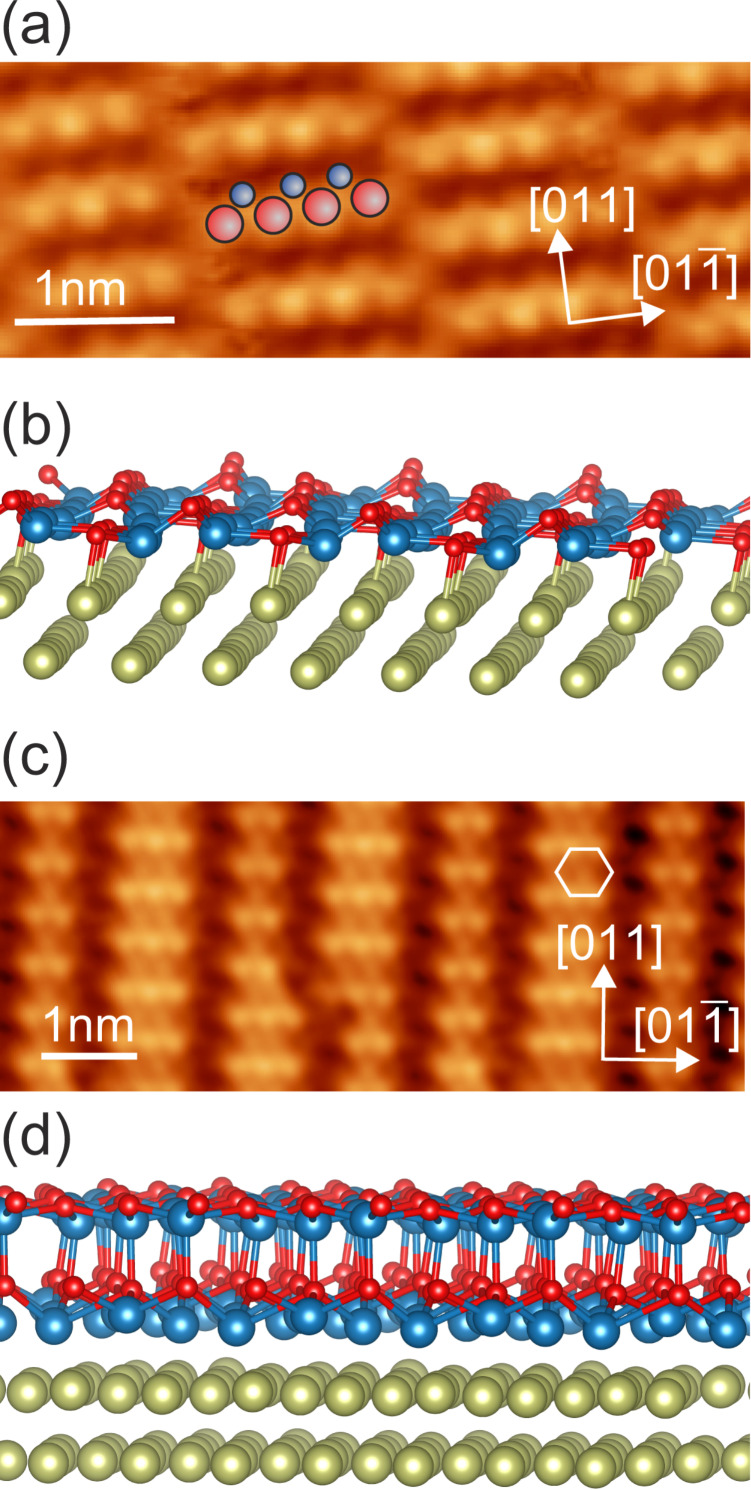
(a) STM image of the 1BL CoO film on Ir(100) with a corrugation of 100 pm and (b) side view of the relaxed film structure from a DFT calculation viewed approximately along the 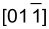 direction of Ir(100). (c) STM image of the 2BL CoO with a corrugation of 20 pm and (d) side view of the film structure. In both cases the terminating oxygen atoms are imaged by STM. They form a quasi-hexagonal pattern in (c). Note that the color scaling of (c) is enhanced with respect to (a). The different corrugations of the cobalt oxide films are also reproduced by the calculations. Cobalt, oxygen, and iridium are represented by blue, red, and yellow balls respectively. In (a) and (c) the crystallographic axes of the Ir(100) substrate are shown. Imaging parameters: (a) *U* = 0.1 V, *I* = 0.1 nA; (b) *U* = 0.5 V, *I* = 0.5 nA.

All experiments were performed in ultra-high vacuum (UHV) at a base pressure of 8 × 10^−11^ mbar. STM images were taken using a custom-built low-temperature UHV STM operated at liquid-nitrogen temperature (80 K). We used etched tungsten tips and the bias voltage is the potential of the sample with respect to the tip. Co-DPP (**1**, PorphyChem SAS, 98% purity, Figure 1a) and 2H-TCNPP (**2**, Porphyrin Systems, 97% purity, Figure 1b) were evaporated from a graphite crucible effusion cell. Co-DPP was evaporated at a measured cell temperature of 540 K and 2H-TCNPP at 640 K, which resulted in a molecular deposition rate of 0.04 and 0.06 nm^−2^·min^−1^. Both molecules were carefully outgassed for 2 to 5 h prior to deposition at ±10 K of the evaporation temperature.

CoO was prepared on an Ir(100) single crystal surface cleaned by ion sputtering and annealing. The Ir(100)-(1 × 1) surface was prepared according to [31]. We employ thin films of two distinct thicknesses, which we state in bilayers, derived from the Co–O bilayer of a rock salt CoO(111) crystal. Cobalt was evaporated from a metal rod using an e-beam evaporator. A one-bilayer (1BL) or a two-bilayer (2BL) film were obtained by depositing 0.9 ML or 1.8 ML, respectively, of cobalt on the Ir(100)-(1 × 1) at 320 K substrate temperature followed by annealing in 2 × 10^−9^ mbar O_2_ at 520 K. To improve ordering, the films were flash-heated to 670 K in UHV. The cleanliness, quality and thickness of the prepared substrates was verified by comparison to low-energy electron diffraction intensity data of earlier studies [18,20].

A 1BL CoO(111) film exhibits a corrugated, slightly distorted cobalt oxide bilayer (Figure 2a,b). It forms a one-dimensional moiré on the Ir(100) surface with perfect row matching resulting in a superstructure between *c*(8 × 2) and *c*(10 × 2) with respect to the Ir(100) surface lattice [20]. While the cobalt ions bind directly to the iridium substrate, there is a considerable buckling of 100 pm of the oxygen atoms. The 2BL CoO(111) film is structurally much flatter with a surface corrugation of only 20 pm [18] (Figure 2c,d). From the structural analysis of thicker CoO(111) films we imply that the surface is oxygen-terminated and shows a wurtzite-type of surface termination [19]. This has been confirmed by surface X-ray diffraction analysis of the 2BL film [21]. Although the 2BL film is structurally close to a *c*(10 × 2) surface structure it may be considered as a flat, quasi-hexagonal layer with lattice parameter *a*_2BL CoO_ = 3.0–3.1 Å [18]. STM images of both films show the positions of the oxygen atoms as bright protrusions [20].

## Computational Methods

Non-magnetic ab initio calculations were performed using the Vienna Ab-initio Simulation Package (VASP) [32] employing the PBE-PAW general gradient approximation [33]. To account for dispersion forces the zero damping DFT-D3 correction of Grimme et al. was used [34]. Slabs were constructed from two layers of iridium and one or two bilayers of cobalt oxide. For the iridium lattice the relaxed DFT-D3 parameter (*a* = 3.835 Å) was used. The lateral size was eight *c*(10 × 2) unit cells for the 1BL system (27.1 × 21.7 Å^2^) and eight *c*(8 × 2) unit cells as approximation for the 2BL system ((21.7 Å)^2^). These cell sizes ensured that the distance between repeated images of the molecules was always larger than 4.1 Å in any molecular orientation. The slabs were separated by approximately 12 Å of vacuum (measured from the topmost atom of the adsorbed molecule), resulting in a third cell dimension of 23 Å and 25 Å, respectively. For structural relaxation all molecular atoms were allowed to relax. Only in the case of **1**, the central Co atom was kept fixed laterally at a position guided by the analysis of our experimental STM images of **1** and by our results on Co phthalocyanine [35]. For the 1BL system, only the bottom Ir layer was kept fixed. For the 2BL calculations, the bare substrate was relaxed first, keeping the bottom Ir layer fixed. During subsequent adsorption studies the molecule and the first CoO bilayer were allowed to relax. Calculations were carried out with an energy cutoff of 400 eV and at the gamma point only, all structures were relaxed until forces were smaller than 0.1 eV/Å. STM simulations were performed employing the Tersoff–Hamann approximation [36]. The projected density of states (PDOS) was calculated using the same parameters. To compare the molecular PDOS on the oxide to that on the bare Ir(100), Co-DPP was put on a bridge site on three layers of Ir. The molecule and two layers of Ir were allowed to relax but no further search for the lowest-energy configuration was carried out.

## Results

**Figure 3:**
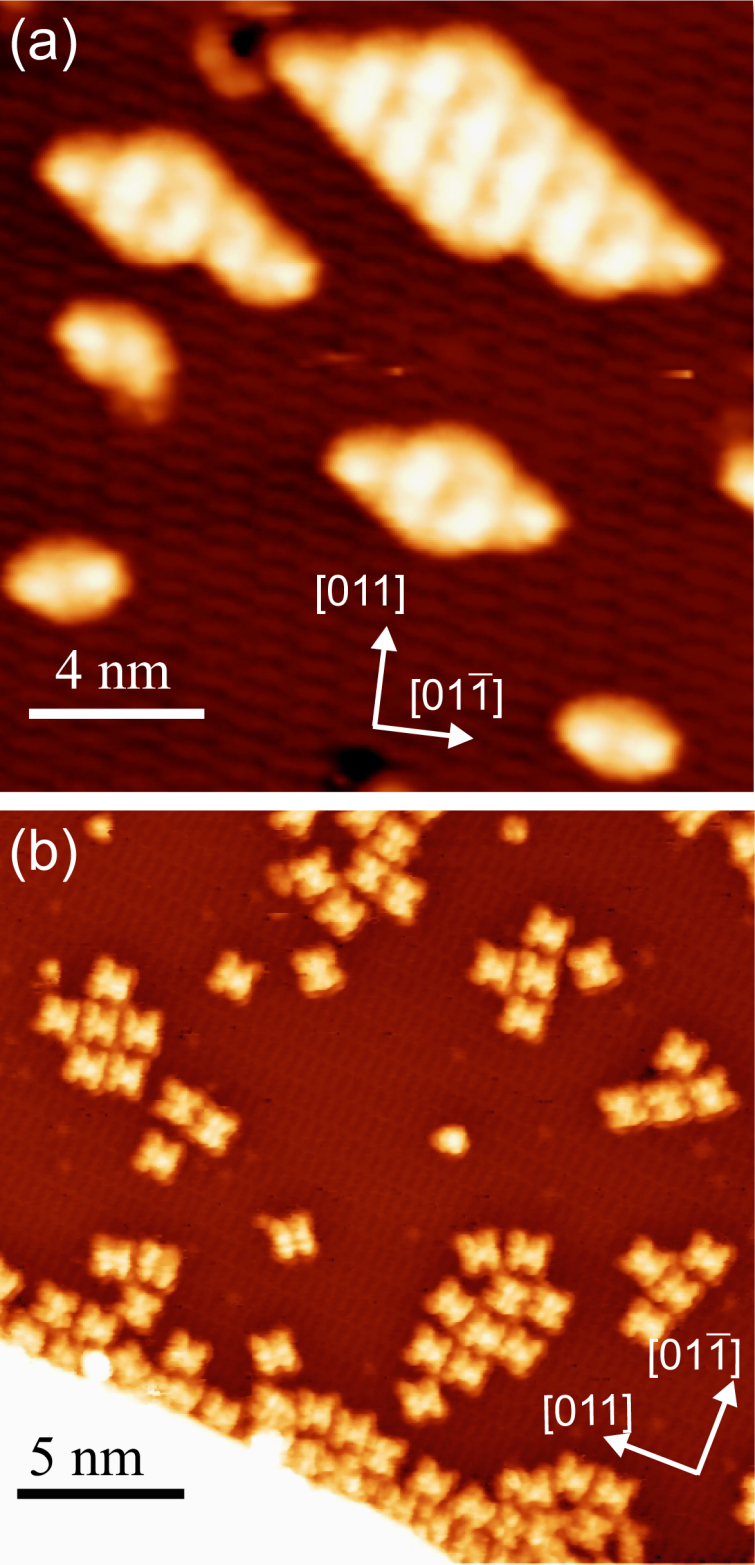
Low coverage of (a) **1** and (b) **2** deposited on 1BL CoO on Ir(100). **1** was deposited at 220 K substrate temperature and annealed to 300 K while for **2** the substrate temperature during deposition was 320 K. Imaging parameters (a) *U* = −1.6 V, *I* = 0.35 nA, apparent molecular height Δ*z* = 3.7 Å; (b) *U* = 2.0 V, *I* = 0.30 nA, Δ*z* = 3.3 Å.

Figure 3 demonstrates the different situations encountered after depositing **1** or **2** on the ultra-thin 1BL CoO film. To activate surface diffusion of the molecules, a temperature of 300–320 K was applied to both systems either by keeping the substrate at that temperature during deposition or by short time (5 min) annealing. Either choice of thermal treatment resulted in the same molecular structures. Higher temperatures could not be applied to **2** without changing the appearance of the molecules. This is attributed to metalation of the free-base porphyrin with Co ions [12–13]. Both molecules adsorb flat-lying on the cobalt oxide film. While **1** forms compact well-ordered islands even at low molecular coverage (Figure 3a), **2** tends to agglomerate into small groups in which neither long-range order nor a preferred binding motive can be detected (Figure 3b). This latter observation remains true for larger coverages at which the size of such agglomerates is increased. The situation for **2** is comparable to adsorption properties observed on Cu(111) when metal-organic coordination is not activated [10]. However, different to Cu(111), even annealing to 420 K – and, in consequence, metalating – does not improve the molecular ordering of **2** on 1BL CoO. Further heating cannot be applied since oxidation of the molecules sets in.

**Figure 4:**
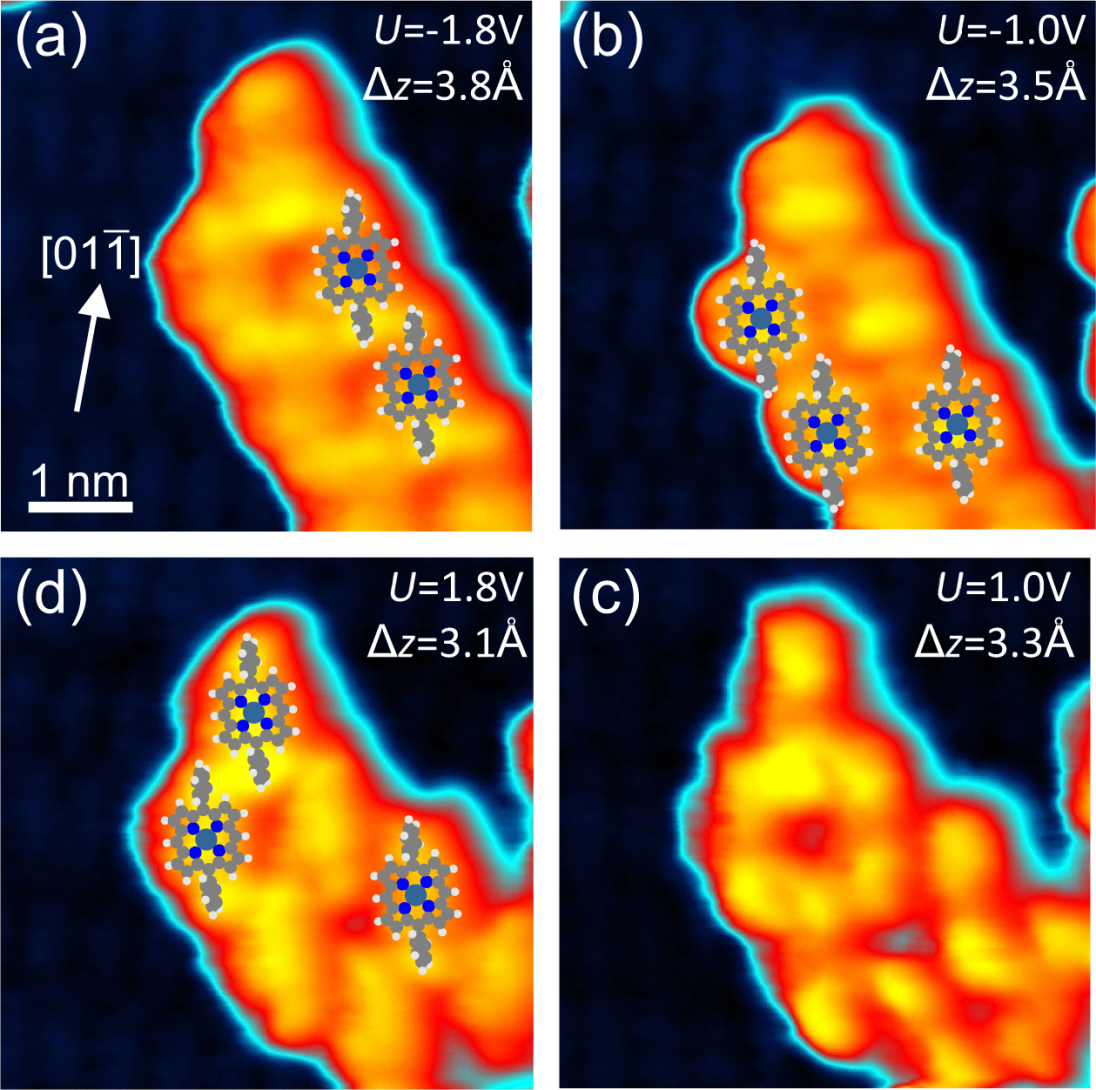
(a–d) Sequence of voltage-dependent STM images of a self-assembled island of **1** on 1BL of CoO. (a) and (b) represent filled-state images while (c) and (d) are empty-state images. The tunneling current for all images was *I* = 0.35 nA. The apparent molecular height is indicated in the panels. All images have the same lateral scale and orientation (see (a)), the color scale is non-linear for better visibility. Due to unwanted tip–molecule interactions the assembly changed between (b) and (c).

The self-assembled structures of **1** may be identified in detail with the help of bias-dependent STM imaging, which allows for an unambiguous identification of single molecules in the structure (Figure 4). The resulting commensurate assembly pattern is indicated by pairs of molecules. While in Figure 4a and Figure 4c the phenyl group becomes dominant in the STM images, it is almost invisible in Figure 4b and a homogeneous contrast develops in Figure 4d. Such compact assemblies were previously observed for the case of the non-metalated 2H-DPP compound on Ag(110) [24] and on Au(111) [22], but, for example, not on Cu(111) [23]. For the assembly on 1BL CoO, the distance between the phenyl rings is a little larger than 2*a*_Ir_ = 5.4 Å, which indicates that this cannot provide a substantial attractive interaction. A distance of approx. 3 Å is found between a phenyl group and the macrocycle of a neighboring molecule in the assemblies of Figure 3a and Figure 4. This could provide an attractive interaction between the involved aromatic systems. Similar values are found on the metal surfaces. The details of these compact structures on different substrates are therefore regulated by the substrate lattice. Furthermore, similar to what is observed for many metal substrates, the central metal ion of the molecule is not imaged as a protrusion on CoO for tunneling bias voltages of ±2.0 V around the Fermi energy.

**Figure 5:**
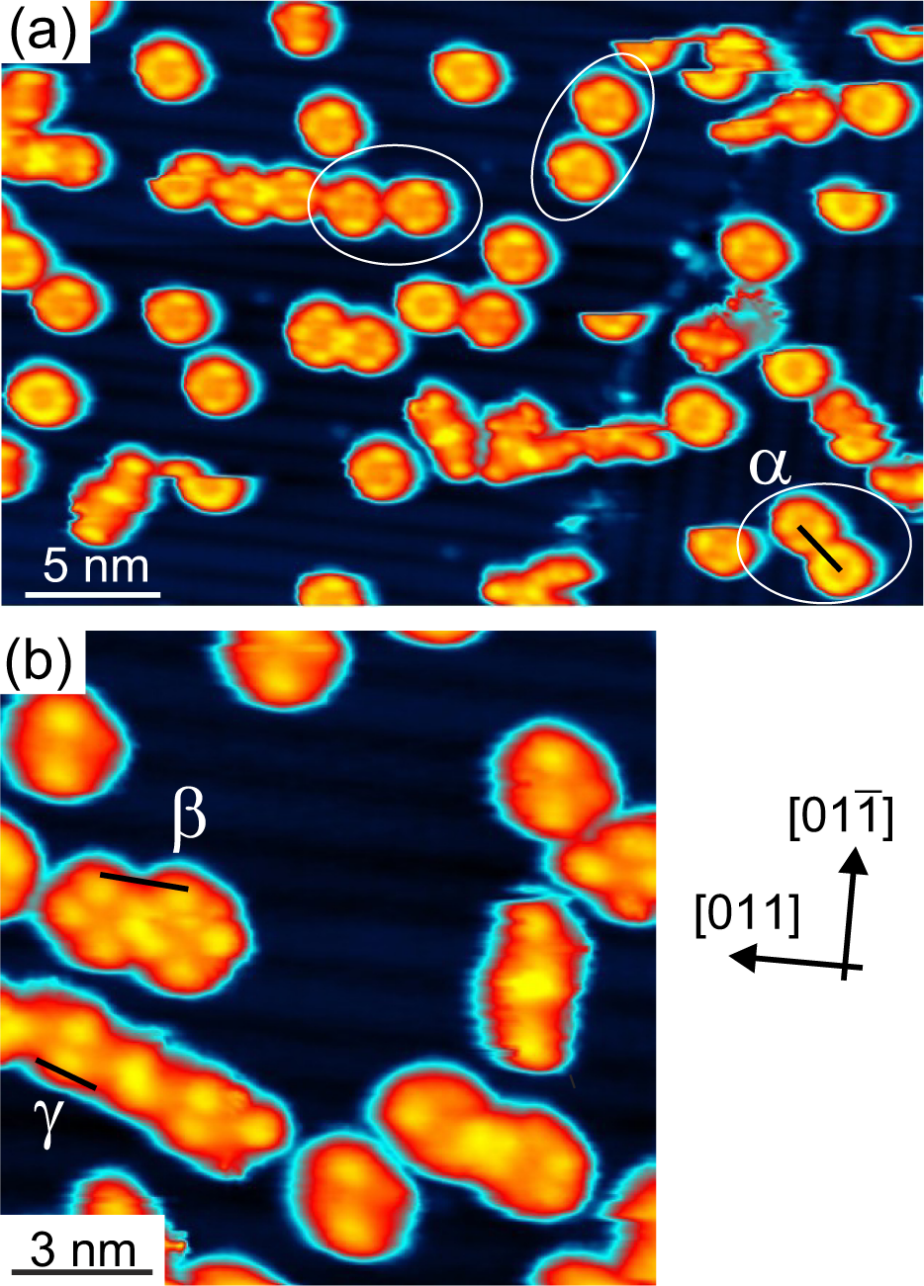
(a) **1** on 2BL CoO deposited at 200 K and imaged at 80 K. The molecules appear as round features unless close to a neighboring molecule or substrate defect. This indicates that **1** rotates around its central Co ion at time scales that are fast with respect to the STM scanning time of a few milliseconds. The STM image represents a time average and shows that the potential landscape of the rotating molecule has two minima with an angle of approximately 90° between each other. The intermolecular distance of the encircled pairs indicated by the black bar at one of them is α = 2.6 nm or larger. (b) Detail of the intermolecular interaction. In close proximity the interaction between neighboring molecules stops the rotation. While the pair β still rotates, molecules γ are stationary. The distances are β = 1.9 nm and γ = 1.5 nm. Imaging parameters *U* = 1.8 V , *I* = 0.15 nA, apparent molecular height Δ*z* = 3.3 Å. (Color scale is non-linear for better visibility.)

In Figure 5 we analyze the behavior of **1** on the 2BL CoO film. Imaging of **1** proved difficult at liquid-nitrogen temperatures since the molecules are easily pulled by the STM tip even at junction resistances of *R_T_* = 10 GΩ. When molecules remain in place, they are imaged as round doughnut-like objects with four positions along the ring, which are imaged brighter (Figure 5a). By manipulating the molecules with the STM tip, we almost never detected a defect in the oxide layer at which the molecules might have been pinned. Only if molecules come close to each other or are trapped by a defect does the former dumbbell shape reappear proving that no change of the molecular structure has taken place (Figure 5b). Hence, the round appearance of the molecules is due to a rotational motion much faster than the imaging speed of the STM, which is in the millisecond regime. The STM images, therefore, represent time averages of the motion of the molecule. The positions where the ring is imaged brighter corresponds to positions that the molecule is more likely to be found in. Such a rotation around a central axis perpendicular to the surface has previously been observed for variety of molecules [37–39]. Many metal phthalocyanines have been shown to rotate on metal surfaces [40–43], but notably also a modified Co phthalocyanine on Si(100) [44].

Molecules sometimes come close enough to each other or can be manipulated such that the rotational motion first gets modified due to the interaction between the phenyl rings of the molecule and finally stops. At a molecule–molecule distance of 2.6 nm and larger (encircled in white in Figure 5a), the appearance in STM of a molecule is not, or only slightly, modified by its neighbors. At a distance of 1.9 nm (β in Figure 5b) the configuration where the molecules attach in a chain-like fashion is slightly more probable, that is, it is imaged brighter in STM. However, the bond still breaks and the molecule may rotate or swing between its various orientations. Only if the intermolecular distance is reduced further to 1.5 nm (γ in Figure 5b) the molecules eventually assemble in stable chain-like structures. It can be expected that such structures will form at larger molecular coverage due to steric blocking of the rotational motion. We propose that the rotating molecular state at temperatures of 80 K and above prevents the molecules from locking into a compact self-assembled layer at low coverage. When stationary molecules with a rotational potential barrier larger than the diffusion barrier self-assemble, the intermolecular forces guide the molecules to diffuse into a state of low-energy at an appropriate bonding distance. This is, for example, observed at low coverage of 2H-DPP on Cu(111) [23]. When, as it appears here, the diffusion and the rotational barrier are of equal magnitude, the intermolecular forces cannot exert their directional influence on the diffusive motion of the molecules since the directional intermolecular bond always “swings open”. Increasing the annealing temperature does not help to form supramolecular structures at low coverage. At 300 K, all molecules will attach to defects or move to 1BL CoO areas present in the film.

**Figure 6:**
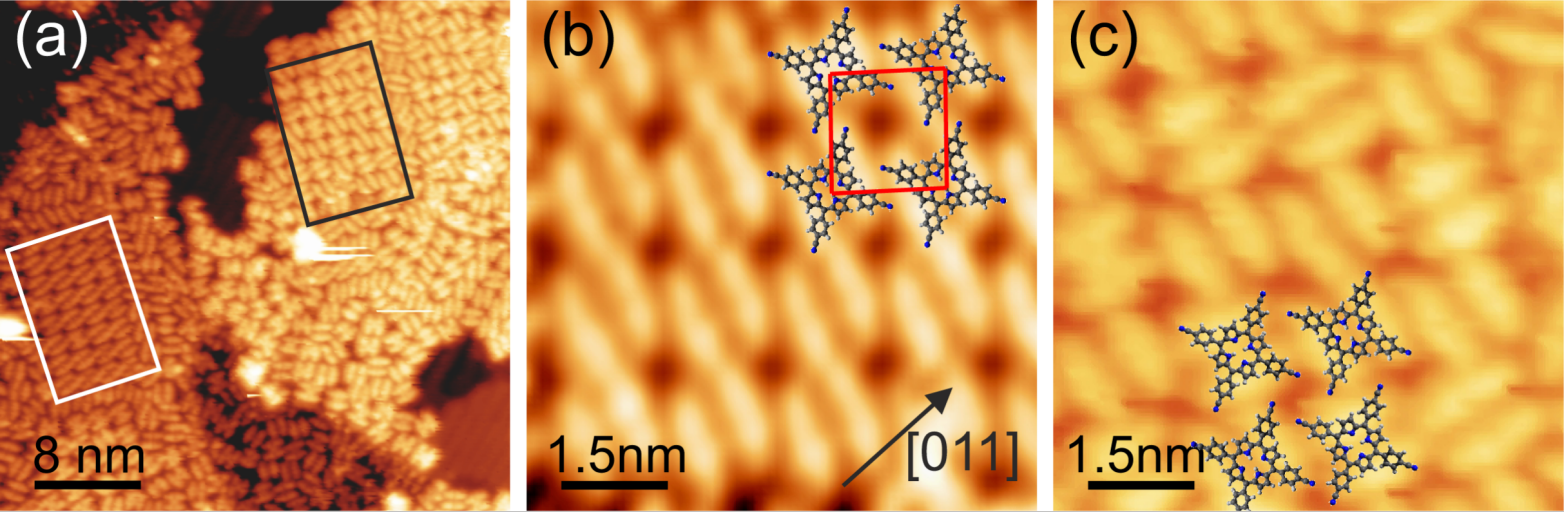
(a) **2** on 2BL CoO deposited at 300 K and imaged at 80 K. The molecules assemble into compact ordered islands with different interaction patterns. (b) Detailed view of the area in (a) marked in white and suggested arrangement of **2** within the unit cell (red). Here, dipolar interactions and hydrogen bridge bonds are observed. (c) The same as (b) but for the area marked in black. Here, only hydrogen bridge bonds are active. Imaging parameters *U* = 3.0 V, *I* = 0.1 nA, apparent molecular height Δ*z* = 3.1 Å.

In contrast, **2** forms compact islands on the 2BL film when the molecules are deposited at approx. 300 K or when the system is annealed to that temperature (Figure 6a). The appearance of **2** on the 2BL film is very similar to that on Ag(111) and allows for an identification of individual molecules in the compact structures [10]. Different superstructures are observed, which also differ in the type of intermolecular interactions. The structure marked by a white rectangle in Figure 6a, shown in detail in Figure 6b, contains two bonding motifs. The first is the dipolar interaction of CN groups of neighboring molecules that are oriented parallel to each other. This bond mediates the interaction along the vertical direction in Figure 6b. The second is a hydrogen bond between a CN group and the macrocycle of a molecule nearby (along the horizontal direction in Figure 6b). A structural analysis of Figure 6b reveals a 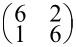 superstructure with respect to the quasi-hexagonal structure of the CoO 2BL film (red rectangle in Figure 6b). Arranging **2** in its gas phase configuration within this cell, we find that the CN groups of the dipolar bond have a distance of 0.4 nm and that the hydrogen bond has a length of 0.3 nm between the N atom and the closest H atom of the macrocycle. Expecting some distortions of the molecule on the surface, this is well in the range of dipolar and hydrogen bond distances [25,30,45]. From the molecular orientation of the assembly marked by the black rectangle in Figure 6a, shown in detail in Figure 6c, it becomes apparent that the dipolar interaction is missing. From that we conclude that the two types of interaction are of almost equal strength, and hence also other compact formations observed display different patterns of these bond types. A similar delicate balance of the interaction strength between functionalized porphyrins has also been observed on metal surfaces [27].

## Discussion

**Figure 7:**
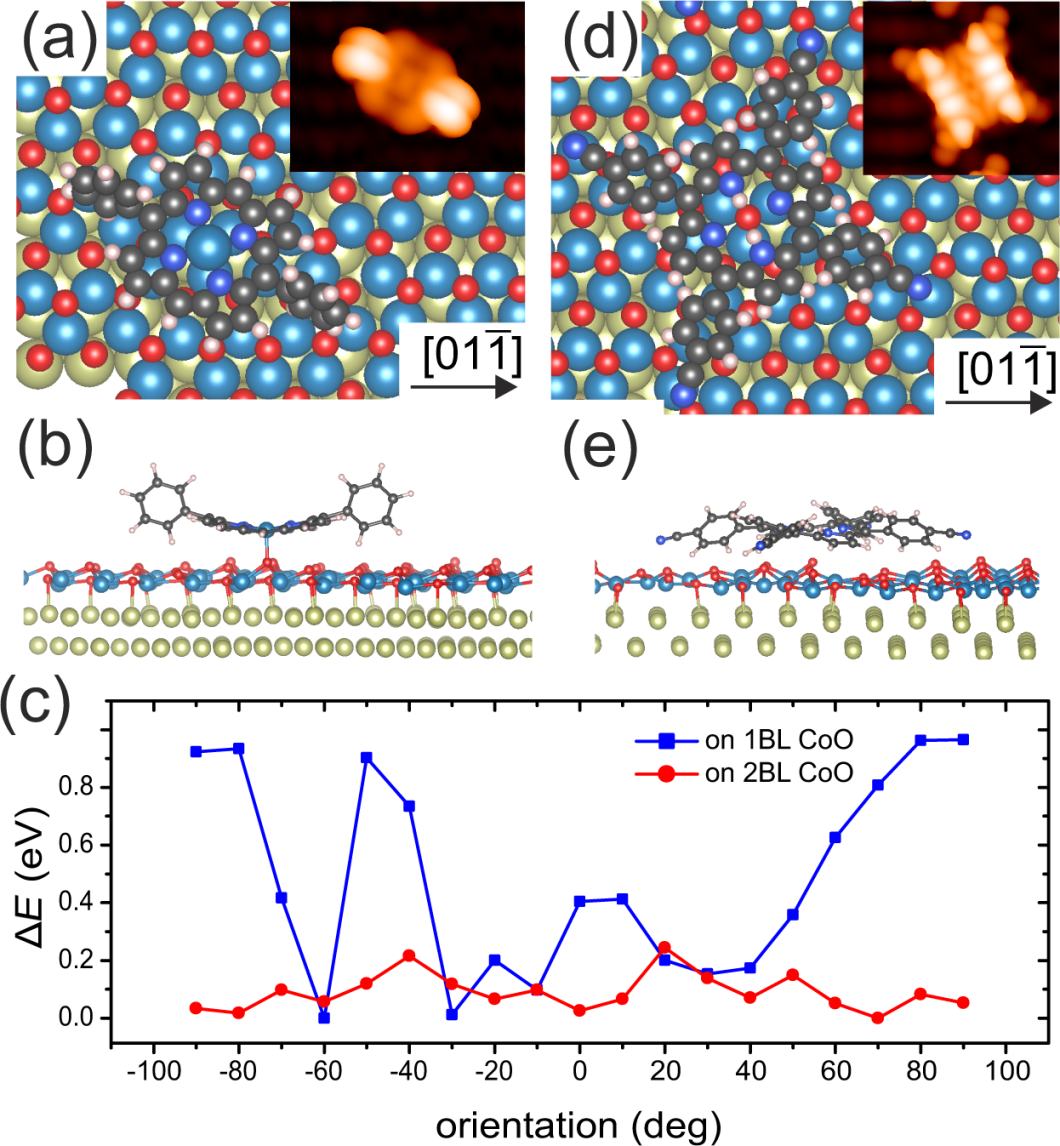
(a,b) Top and side view, respectively, of the relaxed structure of **1** on 1BL CoO according to our DFT calculations. (c) Calculated total energy difference of **1** as a function of the molecular orientation on CoO ultrathin films. Significant rotational barriers of 0.9 eV exist on the 1BL film, while on the 2BL film the activation energy of rotational motion is below 200 meV. The orientation is given as the angle of the molecular axis with respect to the 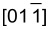 direction of the Ir(100) surface. (d, e) Top and side view, respectively, of **2** on 1BL CoO. Insets in (a) and (d) are STM-DFT simulations at bias voltages of −1.6 V and +2.0 V, that is, the voltages at which the images of, respectively, Figure 3a and Figure 3b.

To gain insight into the reasons behind the experimental findings, DFT calculations were performed. Both, **1** and **2** were structurally relaxed on the 1BL CoO film in order to find the adsorption geometry. The lowest-energy configurations of **1** are shown in Figure 7a,b. We find that the central Co atom of **1** forms a strong bond with one of the O atoms of the substrate (Figure 7b). The Co–O bond length is 1.92 Å and the bonding O is lifted from its original position by 0.2 Å. This is essentially identical to the situation found for Co phthalocyanine on the same surface [35]. The only difference is that the Co atom of **1** stays in the plane of the porpyhrin macrocycle. For Co phthalocyanine, we observe lateral and vertical distortions of up to 0.25 Å of the CoO substrate layer upon adsorption of **1**. However, the repulsive interaction between the two phenyl rings and the surface causes the molecule macrocycle to bend considerably. The largest surface height difference between carbon atoms of the macrocycle amounts to 0.8 Å. The calculated adsorption energy is *E*_ads_ = −(*E*_total_ − *E*_CoDPP_ − *E*_CoO/Ir_) = 2.7 eV. The corresponding STM simulation (Figure 7a, inset) agrees well with the symmetric dumbbell appearance in the experimental images.

We also calculated the relaxed structure of **1** on 2BL CoO, where the molecule adopts a ruffled configuration [46]. We find that the maximum surface height difference between carbon atoms of the macrocycle now amounts to only 0.5 Å while the bond length of the Co atom of **1** to the surface oxygen is now 3.0 Å. This means the distance between the molecule and the surface corresponds closely to the sum of the van der Waals radii of carbon and oxygen (3.2 Å). The binding energy is reduced to *E*_ads_ = 1.55 eV. It was attempted to find a local energy minimum in an adsorption state much closer to the surface but the calculation returned to the weakly bound state. This much weaker binding and larger molecule–surface distance also manifests itself in greatly different energy barriers with respect to the rotation of the molecule on the surface. To this end, we calculated the total energy for different orientations of **1** on 1BL and 2BL CoO (Figure 7c). The orientation is given as the angle between the molecular axis (defined by the phenyl groups) and the 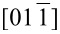 direction of the Ir substrate. While **1** on 1BL experiences barriers up to 0.9 eV, which effectively hinder rotation at 80 K, on 2BL the potential landscape is rather flat, with two maxima representing barriers of only approx. 200 meV.

The configuration of **2** on 1BL CoO is shown in Figure 7d,e. The molecule adopts a saddle-shape configuration. Due to the absence of molecular self-assembly on the 1BL film, it was suspected that the cyanophenyl rings would form bonds to the substrate. Extensive tests were performed to ensure that the found configuration captures the interaction with the substrate correctly. We started (due to simplicity) with a relaxation based on the configuration of **1** with the central Co atom replaced by two H atoms and two cyano groups attached to the phenyl rings of **1**, that is, a 2*H*-dicyanophenylporphyrin. Upon relaxation the macrocycle flattened and the cyanophenyl rings reduced their tilts from the almost orthogonal configuration towards a 45° tilting angle. This indicated an energy gain upon flattening of the molecule and allows for an interaction between the cyano group and the substrate. Consequently, we tested the relaxation of **2** in several attempts starting with configurations where the phenyl rings were bent such that a bond between the cyano group and a Co ion of the substrate could be formed. However, the structure always relaxed into a state in which the N–Co distance exceeded 3.1 Å. While the STM simulation of that configuration shown in Figure 7d matches well with some of the molecules depicted in Figure 3b, it cannot be excluded that **2** assumes different configurations on the 1BL film that are not captured by the present state of modeling. Nevertheless, the weak bending of the phenyl rings (approx. 5°) indicates a molecule–substrate interaction that is absent in the case of **1**. We note that the superstructure formed by **2** on 2BL CoO (Figure 6) is not commensurate to the 1BL film. Hence, the partial engagement of the cyano groups in a surface bond may weaken the intermolecular forces and thereby strengthen the regulating influence of the substrate lattice. In consequence, the formation of any commensurate or incommensurate self-assembly structure is prevented on 1BL CoO.

**Figure 8:**
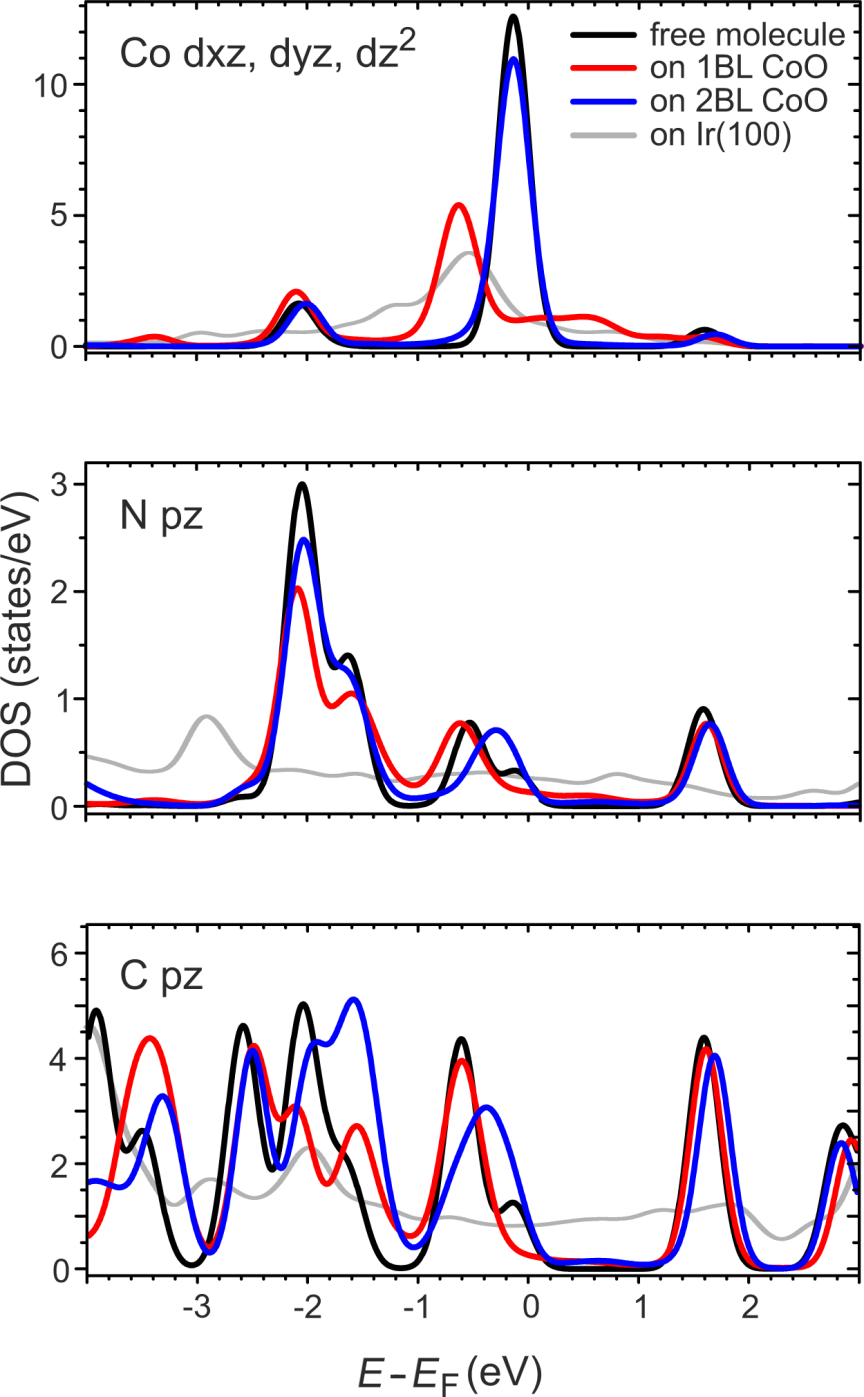
Calculated projected density of states (PDOS) near the Fermi energy of molecular orbitals with components parallel to the surface normal.

Finally, to investigate the degree of electronic decoupling, in Figure 8 we compare the PDOS for the free molecule of **1** and its adsorbed state on 1BL, 2BL CoO, and on Ir(100). The PDOS on Ir(100) shows significant broadening of the molecular orbitals due to a strong hybridization with the Ir 5d and 6s states. In contrast, this hybridization is absent for the 1BL and 2BL CoO system. On 1BL CoO there is a strong hybridization of the molecular Co d orbitals with the states of the substrate. Consequently, significant changes are observed. However, already for the N atoms that are bound to the molecular Co atom this influence is reduced considerably. The comparison of the PDOS of the free molecule with that adsorbed on 2BL CoO shows that the Co PDOS is the same as that of the free molecule, indicating a weak hybridization of the Co center atom with the substrate. The remaining deviations between the N and C PDOS of **1** adsorbed on 1BL or 2BL CoO and that of the free molecule are difficult to interpret. They are surely a sign of weak hybridization with the CoO film, maybe in combination with an influence of the different molecular conformations.

We interpret these findings as a demonstration that the oxide effectively decouples the molecules from the metal substrate and that their electronic structure remains almost undisturbed when adsorbing on the CoO films. Certainly, due to the neglect of spin and correlation in the calculations, the true PDOS will deviate and conclusions have to be drawn with care. However, the work function of CoO is, with 5.85 eV (1BL) and 6.01 eV (2BL) [47], significantly higher than that of most metals. Following the arguments of Yang et al. [48], a charge transfer into unoccupied molecular orbitals is not expected and one can therefore consider the molecules not only to be physically but also electronically decoupled from the metal substrate at least for a CoO thickness exceeding 1BL.

## Conclusion

We investigated in detail the adsorption behavior of a non-functionalized, metalated phenylporpyhrin (Co-DPP, **1**) and a cyano-functionalized, non-metalated phenylporpyhrin (2H-TCNPP, **2**) on cobalt oxide CoO(111) films in the ultrathin limit of one and two bilayer thickness. It is found that the molecule–substrate interaction decreases with increasing cobalt oxide thickness. Nevertheless, dispersion forces constitute a considerable binding potential, which keeps the molecules in a flat adsorption geometry. With respect to self-assembly of the molecules it is found that in the case of Co-DPP on the one-bilayer cobalt oxide film compact self-assembled islands are formed. This is due to the potential landscape provided by the ultrathin film, which supports the self-assembly despite the rather weak intermolecular interaction mediated by the phenyl groups. The surface influence is absent or considerably weaker when two bilayer films are deposited. Here, the Co-DPP rotate at temperatures of 80 K and higher, effectively preventing self-assembly. Conversely, the corrugation of the potential-energy landscape for the cyano-functionalized porphyrin prevents self-assembly on the one bilayer film, but the strong intermolecular interaction creates compact islands on the smoother two bilayer film. Calculations of the projected density of states of **1** on the bare metal and on the oxide layers demonstrate that also a high degree of electronic decoupling from the metal substrate is achieved at the cost of only weak hybridization with the oxide. The work therefore shows that the subtle interplay within the hierarchy of energies leading to self-assembly is also active on oxide surfaces and that the oxygen-terminated CoO(111) surface can be used as a decoupling layer for molecular studies.
